# The NORTh Europe HOStile access TAVI (NORTHOSTAVI) registry

**DOI:** 10.3389/fcvm.2025.1674218

**Published:** 2025-09-26

**Authors:** Joelle Kefer, Yusuke Kobari, Rachid Briki, Christophe Dubois, Frederic De Roeck, Emmanuel De Cock, Charles Pirlet, Adel Aminian, Julie Colas-Florial, Maarten Vanhaverbeke, Antoine Guédès, Yoann Bataille, Amaury Sogorb, Kees Van Bergeijk, Céline Bugli, Frederic Maes, Johan Bosmans, Ian Buysschaert, Liesbeth Rosseel, Bert Vandeloo, Joanna Wykrzykowska, Ole De Backer

**Affiliations:** ^1^ Division of Cardiology, Cliniques Universitaires Saint-Luc et Pôle de Recherche Cardiovasculaire, Institut de Recherche Expérimentale et Clinique (IREC), Université Catholique de Louvain (UCLouvain), Brussels, Belgium; ^2^ The Heart Center, Righospitalet, Copenhagen, Denmark; ^3^ CHU Saint-Pierre, Brussels, Belgium; ^4^ Gasthuisberg, UZ Leuven, Leuven, Belgium; ^5^ UZ Antwerpen, Antwerpen, Belgium; ^6^ AZ Sint-Jan Campus, Brugge, Belgium; ^7^ CHR Citadelle, Liège, Belgium; ^8^ Division of Cardiology, Centre Hospitalier Universitaire de Charleroi, Charleroi, Belgium; ^9^ Hartcentrum Aalst, AZORG, Aalst, Belgium; ^10^ Hartcentrum AZ Delta, Roeselare, Belgium; ^11^ CHU UCL Namur- Mont Godinne, Yvoir, Belgium; ^12^ Jessa Ziekenhuis, Hasselt, Belgium; ^13^ UZ Brussel Jette, Brussels, Belgium; ^14^ University Medical Center, Groningen, Netherlands; ^15^ SMCS (Plateforme Technologique de Support en Méthodologie et Support Statistique), UCLouvain, Louvain-la-Neuve, Belgium

**Keywords:** TAVI, endovascular access, alternative access, peripheral arterial disease, VARC-3

## Abstract

**Background-aims:**

Comparisons between alternative access routes for transcatheter aortic valve implantation (TAVI) in patients with hostile vascular access are scarce. This study aimed to perform a head-to-head comparison of various techniques (percutaneous transaxillary [P-TAx], surgically-assisted transaxillary [S-TAx], transcarotid [TCar], percutaneous transluminal angioplasty-assisted transfemoral [PTA-TF], transbrachiocephalic [TBra], and transcaval [TCav]) with respect to the 30-day outcome of patients undergoing TAVI.

**Methods:**

NORTHOSTAVI (*N*ORTh Europe HOStile access TAVI) was an international registry that included patients undergoing TAVI via various endovascular transarterial access routes in Northern European countries. The primary endpoint was the adjusted 30-day rate of composite overall mortality, disabling stroke, main access site-related major vascular complications, and major bleeding according to VARC-3 criteria.

**Results:**

In total, 531 patients were enrolled in the study across 14 centers. The main access routes were TCar (*N* = 183, 35%), P-TAx (*N* = 153, 29%), S-TAx (*N* = 79, 15%), and PTA-TF (*N* = 76, 14%), while TBra and TCav were used in 23 (4%) and 17 (3%) patients, respectively. Technical success was achieved in 95% of cases, 30-day overall and event-free survival rates were 97% and 91%, respectively, similar between groups. P-TAx, but not S-TAx or PTA-TF, was associated with an increased adjusted risk of overall stroke (adjusted OR: 4.21; 95%CI: 1.129–15.747; *p* = 0.003) compared to TCar. PTA-TF was associated with an increased adjusted risk of main access site-related major vascular complications (adjusted OR: 7.71; 95%CI: 1.367–43.554; *p* = 0.02) compared to TCar.

**Conclusions:**

The NORTHOSTAVI registry showed that in patients with hostile iliofemoral anatomy, TAVI via various endovascular transarterial access route is efficient and safe.

## 1 Introduction

The transfemoral (TF) route is the default access for transcatheter aortic valve implantation (TAVI), showing reduced mortality compared to surgical aortic valve replacement (SAVR) in meta-analysis of randomized trials (1). Approximately 5%–10% of patients who are candidates for TAVI have severe peripheral artery disease that precludes the procedure via this route (2, 3). Transthoracic (transapical and transaortic) access routes have demonstrated higher mortality than TF TAVI, due to the higher-risk profile of the selected patients and the more invasive nature of these procedures (4, 5).

Therefore, several alternative endovascular access routes (transsubclavian, transaxillary, transcarotid, transbrachiocephalic, and transcaval) were developed to mitigate the poor outcome of transthoracic TAVI in patients with hostile iliofemoral anatomy (6–11). Some peripheral angioplasty techniques, including balloon, stent, and lithotripsy, have demonstrated the feasibility of a TF approach despite unfavorable access (12, 13).

The transarterial series showed high technical success with better outcome than transthoracic procedures, yet with higher rates of vascular complications and strokes compared to TF TAVI (14). Nowadays, the selection of the optimal access for patients with hostile iliofemoral anatomy is based on physician preference and/or available local resources. Comparisons of these alternative techniques are scarce.

The aim of the present study was to perform a head-to-head comparison of various endovascular alternative access routes and the TF approach prepared by peripheral angioplasty with respect to the procedural outcome in patients undergoing TAVI, according to the Valve Academic Research Consortium 3 (VARC-3) criteria (15).

## 2 Methods

### 2.1 Population and data collection

NORTHOSTAVI (NORTh Europe HOStile access TAVI) was an international, multicenter registry that included retrospective data on patients undergoing TAVI in Northern European countries (Belgium, Denmark, and the Netherlands). This all-comers registry included patients with hostile femoral access, defined as severe peripheral artery disease precluding the TF access route, who underwent TAVI via an alternative endovascular access or by the TF access assisted by peripheral angioplasty.

Data from patients at participating centers were collected prospectively on site according to the rules of the local ethics committee. De-identified data were entered retrospectively into a dedicated database of the present registry for observational analysis. All items in the database were defined according to the VARC-3 criteria.

Ethical approval was granted by the ethics committee of each participating center.

The inclusion criteria were patients with severe aortic stenosis and severe peripheral artery disease precluding TF TAVI, who underwent alternative endovascular TAVI (percutaneous transaxillary [P-TAx], surgically-assisted transaxillary [S-TAx], transcarotid [TCar], transbrachiocephalic [TBra], transcaval [TCav]) or percutaneous transluminal angioplasty-assisted TF [PTA-TF]), with a 30-day follow-up available, according to the VARC-3 criteria.

Exclusion criteria were transthoracic TAVI, peripheral artery disease not requiring an angioplasty to allow a TF TAVI, and unplanned peripheral angioplasty performed as a bailout treatment due to a vascular complication during the procedure.

### 2.2 Endpoints

The primary endpoint was the adjusted 30-day rate of composite overall mortality, disabling stroke, major bleeding, and main access site-related VARC-3 major vascular complications.

The secondary endpoints were the components of the primary endpoint, the new permanent pacemaker rate, and the intended performance of the valve according to the VARC-3 criteria at 30 days.

### 2.3 Statistical analysis

Continuous variables are presented as mean ± 1 standard deviation when symmetrically distributed, and as median and range when non-symmetrically distributed. Normality was assessed using the Shapiro–Wilk test. Categorical variables are presented as counts and percentages. Continuous variables were compared among groups using ANOVA when normally distributed, and using the Kruskall-Wallis test with *ad hoc post hoc* tests when not normally distributed. Categorical variables were tested using the chi-square test or Fisher's exact test, as appropriate. A multivariate logistic regression analysis was performed to explain the primary endpoint and adjust for confounding factors, including age, Society of Thoracic Surgeons (STS) score, and left ventricular ejection fraction. Estimates of freedom from the composite endpoint of death and major adverse events were obtained using the Kaplan–Meier estimation method. The log-rank test was used to assess the difference in survival curves. A *p*-value < 0.05 was considered statistically significant. The statistical analyses were conducted using IBM SPSS Statistics for Windows, version 29.0.2.0 (IBM Corp., Armonk, NY, USA) and JMP® Pro 17.2.0 (JMP Statistical Discovery LLC, Cary, NC, USA).

## 3 Results

### 3.1 Patients

Between November 2009 and June 2024, 531 patients were enrolled in the study across 14 centers. The four main access routes for TAVI were TCar (183 patients, 35%), P-TAx (153 patients, 29%), S-TAx (79 patients, 15%), and PTA-TF (76 patients, 14%), while TBra and TCav were used in 23 (4%) and 17 (3%) patients, respectively. The number of TAVIs by access route at each participating center is presented in Supplementary Table S1. Five out of 14 centers restricted alternative access to one technique only, while the TBra route was only used in one center.

The population in this study was considered intermediate to high risk: the mean age was 81 ± 7 years, the mean STS score was 5 ± 4, and NYHA class III–IV was observed in 60% of patients, coronary artery disease in 63%, chronic renal failure in 52%, and chronic lung disease in 35%. Obesity and overweight were observed in 22% and 36% respectively. Baseline characteristics are shown in Table 1, revealing significant differences in age and comorbidities between groups, which resulted in statistically significant differences in STS scores by access route. A prior coronary artery bypass graft was less common in the P-TAx group, likely because of the risk of interference between the left internal mammary artery and the sheath introduced into the left subclavian artery. Chronic lung disease was less common in the TCar group, requiring general anesthesia and endotracheal intubation.

**Table 1:** Baseline characteristics.

Characteristic		Total	TCar	P-TAx	S-TAx	PTA-TF	TBra	TCav	*p*-value
		*N* = 531	*N* = 183	*N* = 153	*N* = 79	*N* = 76	*N* = 23	*N* = 17	
Age, years	Mean ± sd	81 ± 7	83 ± 7	79 ± 7	79 ± 8	81 ± 6	77 ± 9	79 ± 8	<0.0001
Male gender	*n* (%)	317 (60)	115 (63)	88 (57)	27 (34)	40 (53)	14 (61)	8 (47)	0.482
Body mass index, kg/m^2^	Mean ± sd	26 ± 5	26 ± 5	26 ± 6	26 ± 5	26 ± 5	27 ± 5	26 ± 5	0.727
Society of Thoracic Surgeons score	Mean ± sd	5 ± 4	6 ± 5	4 ± 3	4 ± 3	5 ± 3	9 ± 5	5 ± 3	<0.0001
NYHA functional class III-IV	*n* (%)	314 (60)	88 (48)	87 (57)	58 (73)	49 (64)	18 (78)	14 (81)	0.001
Aortic valve area, cm^2^	Mean ± sd	0.7 ± 0.2	0.7 ± 0.2	0.7 ± 0.2	0.7 ± 0.2	0.7 ± 0.2	0.7 ± 0.2	0.7 ± 0.2	0.803
Mean transvalvular gradient, mmHg	Mean ± sd	44 ± 14	45 ± 13	44 ± 15	43 ± 16	44 ± 13	39 ± 14	43 ± 17	0.358
Left ventricular ejection fraction, %	Mean ± sd	54 ± 12	57 ± 11	54 ± 12	52 ± 10	51 ± 11	49 ± 13	55 ± 10	0.0003
Aortic regurgitation ≥ moderate	*n* (%)	76 (14)	29 (16)	14 (9)	19 (24)	9 (12)	5 (22)	0	0.331
Atrial fibrillation	*n* (%)	180 (34)	66 (36)	55 (36)	19 (24)	27 (36)	7 (30)	6 (35)	0.905
Coronary artery disease	*n* (%)	332 (63)	126 (69)	80 (52)	39 (49)	18 (78)	15 (88)	54 (71)	<0.0001
Prior myocardial infarction	*n* (%)	113 (21)	45 (25)	21 (14)	23 (29)	13 (57)	0	11 (14)	<0.0001
Prior coronary angioplasty	*n* (%)	194 (37)	59 (32)	59 (39)	24 (30)	34 (45)	10 (43)	8 (47)	0.286
Prior coronary artery bypass graft	*n* (%)	100 (19)	40 (22)	21 (14)	15 (19)	10 (43)	3 (18)	11 (14)	0.024
Chronic lung disease	*n* (%)	185 (35)	45 (25)	66 (43)	35 (44)	8 (35)	6 (35)	25 (33)	0.0023
Diabetes	*n* (%)	156 (29)	51 (28)	50 (33)	24 (30)	25 (33)	4 (17)	2 (12)	0.244
Mediastinal radiation	*n* (%)	15 (3)	7 (4)	2 (1)	2 (2)	2 (3)	2 (9)	0	0.314
Porcelain aorta	*n* (%)	20 (3.8)	7 (4)	5 (3)	4 (5)	3 (4)	1 (4)	0	0.944
Chronic renal failure	*n* (%)	278 (52)	96 (52)	73 (48)	44 (56)	39 (51)	15 (65)	11 (65)	0.567
Prior stroke	*n* (%)	87 (16)	23 (13)	30 (20)	11 (14)	12 (16)	8 (35)	3 (18)	0.093
Prior pacemaker	*n* (%)	44 (8)	19 (10)	14 (9)	1 (1)	7 (9)	1 (4)	2 (12)	0.575
Prior surgical aortic valve replacement	*n* (%)	17 (3)	7 (4)	5 (3)	4 (5)	1 (1)	0	0	0.597
Prior transcatheter aortic valve implantation	*n* (%)	1 (0.2)	1 (1)	0	0	0	0	0	0.753

P-TAx, percutaneous transaxillary; S-TAx, surgically-assisted transaxillary; TCar, transcarotid, PTA-TF, percutaneous transluminal angioplasty-assisted transfemoral; TCav, transcaval; TBra, transbrachiocephalic.

### 3.2 Procedural outcome

The procedural outcome is detailed in Table 2.

**Table 2:** Procedural outcome.

Characteristic		Total	TCar	P-TAx	S-TAx	PTA-TF	TBra	TCav	*p*-value
		*N* = 531	*N* = 183	*N* = 153	*N* = 79	*N* = 76	*N* = 23	*N* = 17	
Aortic annulus perimeter-derived diameter	Mean ± sd	25 ± 2	25 ± 2	25 ± 2	25 ± 2	25 ± 2	26 ± 3	24 ± 2	<0.0001
Aortic annulus perimeter	Mean ± sd	78 ± 8	78 ± 7	78 ± 8	78 ± 7	78 ± 8	80 ± 8	75 ± 5	0.272
Aortic valve calcium score	Mean ± sd	2996 ± 1394	3039 ± 1,383	2,962 ± 1,262	2,883 ± 1,274	3,188 ± 1,467	2,511 ± 1,905	NAV	0.431
Bicuspid aortic valve	*n* (%)	16 (3)	1 (1)	5 (3)	3 (4)	4 (5)	0	3 (18)	0.006
Type of valve implanted									<0.0001
Balloon/self-expanding valve	%	8/92	9/91	1/99	18/82	9/91	0/100	18/82	
CoreValve/Evolut	*n* (%)	321 (60)	162 (89)	62 (41)	46 (58)	26 (34)	23 (100)	2 (12)	
Sapien	*n* (%)	43 (8)	17 (9)	2 (1)	14 (18)	7 (9)	0	3 (18)	
Portico/Navitor	*n* (%)	136 (26)	4 (2)	75 (49)	18 (23)	28 (37)	0	11 (65)	
Acurate	*n* (%)	31 (6)	0	14 (9)	1 (1)	15 (20)	0	1 (6)	
Size of valve implanted	Mean ± sd	29 ± 3	29 ± 3	29 ± 3	29 ± 3	28 ± 4	27 ± 2	29 ± 3	<0.0001
Type of anesthesia									<0.0001
Local	*n* (%)	47 (9)	1 (1)	15 (10)	1 (1)	29 (38)	0	1 (6)	
Conscious sedation	*n* (%)	73 (14)	3 (1)	44 (29)	6 (8)	20 (26)	0	0	
General	*n* (%)	411 (77)	179 (98)	94 (61)	72 (91)	27 (36)	23 (100)	16 (94)	
Pre-dilatation	*n* (%)	317 (60)	48 (27)	126 (82)	44 (56)	64 (84)	22 (96)	13 (76)	<0.0001
Post-dilatation	*n* (%)	92 (17)	10 (5)	36 (24)	21 (27)	18 (24)	0	7 (41)	<0.0001
Surgical cut-down	*n* (%)	285 (54)	183 (100)	0	79 (100)	0	23 (100)	0	<0.0001
Echo-guided puncture	*n* (%)	245 (46)	0	131 (86)	0	76 (100)	0	17 (100)	<0.0001
Closure technique									<0.0001
ProGlide	*n* (%)	46 (9)	0	19 (12)	0	27 (36)	0	0	
Prostar	*n* (%)	2 (0.4)	0	1 (1)	0	1 (1)	0	0	
Manta	*n* (%)	14 (3)	0	9 (6)	0	5 (7)	0	0	
Duct Occluder	*n* (%)	17 (3)	0	0	0	0	0	17 (100)	
Combination of devices	*n* (%)	126 (25)	0	91 (59)	0	36 (47)	0	0	
Covered stent	*n* (%)	40 (7)	0	33 (22)	0	7 (9)	0	0	
Surgical closure	*n* (%)	285 (54)	183 (100)	0	79 (100)	0	23 (100)	0	
Secondary vascular access									<0.0001
Femoral	*n* (%)	425 (80)	166 (91)	89 (58)	70 (89)	63 (83)	20 (87)	17 (100)	
Radial	*n* (%)	100 (19)	17 (9)	62 (41)	5 (6)	13 (17)	3 (13)	0	
Other	*n* (%)	6 (1)	0	2 (1)	4 (5)	0	0	0	
Type of peripheral angioplasty									
Balloon	*n* (%)	2 (0.4)	0	0	0	2 (2.5)	0	0	
Lithotripsy	*n* (%)	74 (14)	0	0	0	74 (97)	0	0	
Stent	*n* (%)	2^a^ (0.4)	0	0	0	2^a^ (2.5)	0	0	
Fluoroscopy time, minutes	Mean ± sd	26 ± 14	14 ± 7	28 ± 17	24 ± 8	29 ± 11	25 ± 8	34 ± 15	<0.0001
Contrast volume, ml	Mean ± sd	116 ± 61	136 ± 88	95 ± 38	107 ± 38	114 ± 57	184 ± 54	140 ± 46	<0.0001
Technical success	*n* (%)	502 (95)	175 (96)	146 (95)	74 (94)	71 (93)	22 (96)	14 (82)	0.42
Length of hospital stay, days	Mean ± sd	6 ± 5	7 ± 4	4 ± 4	6 ± 8	4 ± 4	9 ± 5	5 ± 5	<0.0001

P-TAx, percutaneous transaxillary; S-TAx, surgically-assisted transaxillary; TCar, transcarotid, PTA-TF, percutaneous transluminal angioplasty-assisted transfemoral; TCav, transcaval; TBra, transbrachiocephalic.

^a^ Two stents combined with intravascular lithotripsy.

Patients underwent TAVI using a self-expanding valve in 92% of cases (Corevalve/Evolut: 60%, Portico/Navitor: 26% and Accurate: 6%) and balloon-expanding Sapien valve in 8%, with significant differences in the types of valves implanted depending on the access route: Evolut/Corevalve was implanted in 89% of cases in the TCar group, while the Portico/Navitor platform was used in 49% of cases in the P-TAx group (*p* < 0.0001).

General anesthesia was required for 77% of the study population and 98% of TCar patients, compared to only 36% of PTA-TF procedures.

Pre-dilatation was used significantly less often in the TCar group (27% vs. >50% in all other groups, *p* < 0.0001). Post-dilatation was less frequently needed in the TCar group as well (5% vs. >20% in all other groups, *p* < 0.0001). A surgical cut-down was used in all TCar and TBra cases, but none of the PTA-TF cases. Echo-guided percutaneous vascular puncture was used in 100% of PTA-TF and TCav procedures and 86% of P-TAx procedures. No surgical cut-down access was echo-guided.

Surgical closure of the main access was performed in all S-TAx, TCar, and TBra cases, but in none of the P-TAx and PTA-TF cases. Percutaneous closure was performed using a combination of devices (mainly Angio-Seal + ProGlide) in 59% and 47% of P-TAx and PTA-TF cases, respectively, while a covered stent was needed in 22% and 9% of P-TAx and PTA-TF cases, respectively. The secondary vascular access was primarily femoral (80%), with the highest rate of radial access observed in the P-TAx group (41%).

For the PTA-TF access route, the most common technique for preparing the femoral vessel was intravascular lithotripsy (IVL), employed in 97% of cases, while a regular balloon was used in two cases and a vascular stent was implanted in addition to IVL in two other cases.

A cerebral embolic protection device (CEPD) was used in 42 patients (7.9%): 32 in PTA-TF and 10 in P-TAx group.

At discharge, antithrombotic therapy was single antiplatelet (*N* = 157, 30%), dual antiplatelet (*N* = 156, 30%), anticoagulant (*N* = 76, 15%), combined antiplatelet + anticoagulant (*N* = 126, 25%) and none in one patient.

The fluoroscopy time was significantly shorter in the TCar group (14 ± 7 min vs. 26 ± 14 min in the total cohort, *p* < 0.0001), whereas the iodinated contrast agent volume was the lowest in the P-TAx group (95 ± 38 ml vs. 116 ± 61 ml in the total cohort, *p* < 0.0001).

Technical success was achieved in 95% of the total cohort, with no significant difference between groups. The mean hospital stay was significantly shorter after PTA-TF and P-TAx (4 ± 4 days vs. 6 ± 5 days in the total cohort, *p* < 0.0001).

### 3.3 30-day follow-up

The in-hospital and 30-day overall mortality rates were 2.4% and 2.8%, respectively, with no significant differences between groups (Table 3).

**Table 3:** 30-day follow-up.

Characteristic		Total	TCar	P-TAx	S-TAx	PTA-TF	TBra	TCav	p-value
		*N* = 531	*N* = 183	*N* = 153	*N* = 79	*N* = 76	*N* = 23	*N* = 17	
Primary endpoint	*n* (%)	60 (11)	14 (8)	23 (15)	8 (10)	10 (13)	1 (4)	5 (29)	0.927
Overall mortality	*n* (%)	15 (2.8)	6 (3.3)	4 (2.6)	2 (2.5)	1 (1.3)	1 (4.3)	1 (5.8)	0.904
Overall stroke	*n* (%)	23 (4.3)	4 (2)	9 (6)	6 (8)	4 (5)	0	0	0.138
Disabling stroke	*n* (%)	9 (2)	2 (1)	3 (2)	2 (3)	2 (3)	0	0	0.468
Main access site major vascular complication	*n* (%)	14 (3)	2 (1)	3 (2)	1 (1)	5 (6)	0	3 (18)	0.013
Major bleeding	*n* (%)	23 (4)	4 (2)	13 (8)	3 (4)	2 (3)	0	1 (6)	0.037
Acute kidney injury ≥2	*n* (%)	39 (7)	2 (1)	19 (12)	5 (6)	7 (9)	1 (4)	5 (29)	<0.0001
New pacemaker	*n* (%)	55 (10)	16 (9)	18 (12)	10 (13)	7 (9)	4 (17)	0	0.188
Echocardiographic 30-day follow-up									
Aortic valve area, cm^2^	Mean ± sd	2 ± 1	2.1 ± 0.6	2 ± 0.5	1.9 ± 0.6	1.9 ± 0.6	2 ± 1	1.8 ± 0.3	0.1
Mean transvalvular gradient, mmHg	Mean ± sd	8 ± 4	9 ± 4	8 ± 4	7 ± 4	8 ± 3	8 ± 4	8 ± 5	0.1
Aortic regurgitation ≥moderate	*n* (%)	20 (4)	8 (4)	2 (1)	2 (2)	3 (4)	5 (22)	0	<0.0001
Device success		480 (90)	167 (91)	143 (93)	72 (91)	67 (88)	17 (74)	14 (82)	<0.0001

P-TAx, percutaneous transaxillary; S-TAx, surgically-assisted transaxillary; TCar, transcarotid, PTA-TF, percutaneous transluminal angioplasty-assisted transfemoral; TCav, transcaval; TBra, transbrachiocephalic.

**Figure 1:**
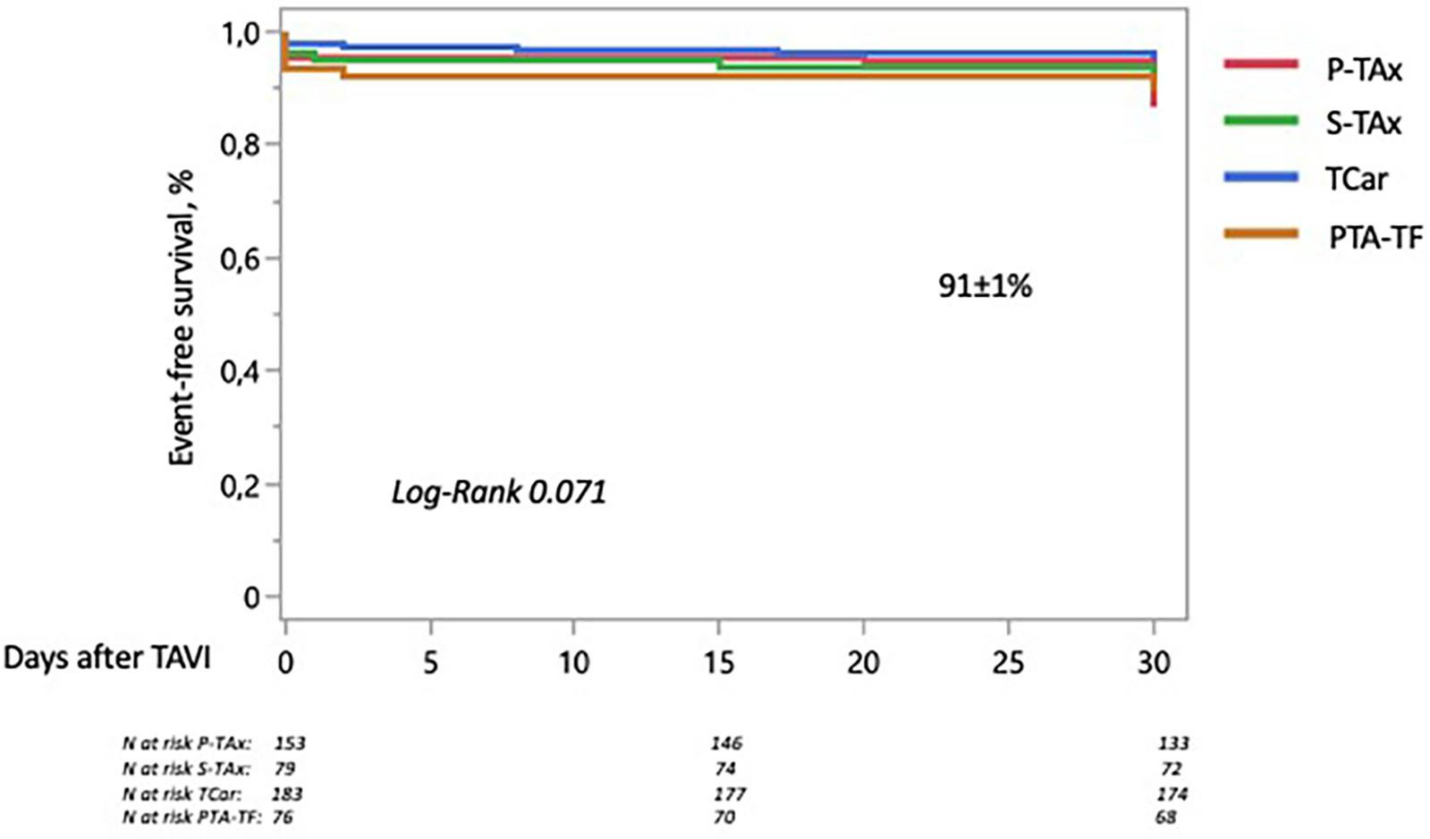
30-day rate of the composite primary endpoint (overall mortality, disabling stroke, major bleeding, and main access site-related major vascular complications) stratified by access route. The Kaplan–Meier analysis showed a 30-day event-free survival rate of 91%, which was similar across groups. P-TAx, percutaneous transaxillary; S-TAx, surgically-assisted transaxillary; TCar, transcarotid; PTA-TF, percutaneous transluminal angioplasty-assisted transfemoral.

Regarding the combined primary endpoint (adjusted 30-day rate of composite overall mortality, disabling stroke, main access site-related major vascular complications, and major bleeding), the Kaplan–Meier analysis showed an event-free survival rate of 91% at 30 days, which was similar across access routes (Figure 1).

**Figure 2:**
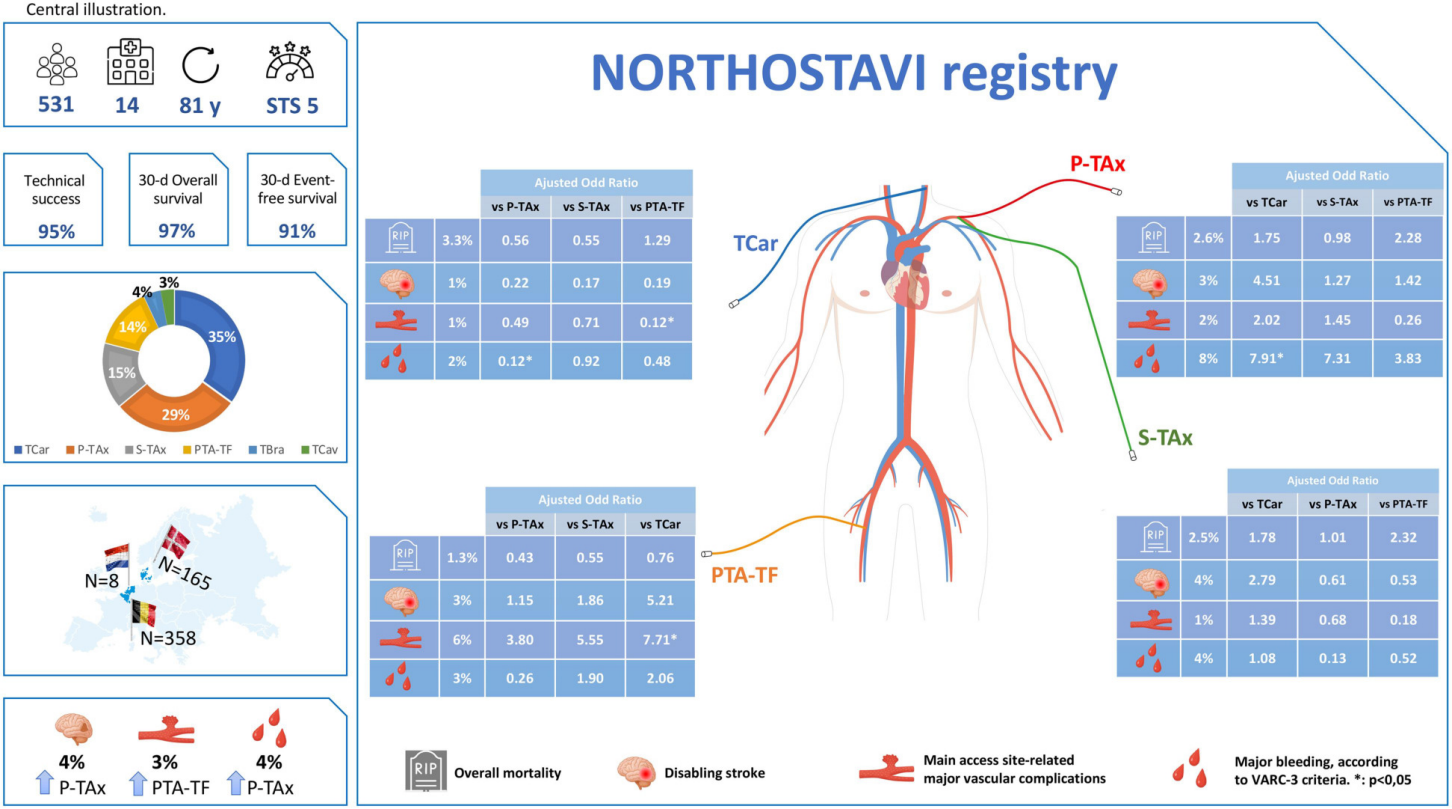
The NORTHOSTAVI registry: 30-day outcome of TAVI by various endovascular transarterial access route. P-TAx, percutaneous transaxillary; S-TAx, surgically-assisted transaxillary; TCar, transcarotid; PTA-TF, percutaneous transluminal angioplasty-assisted transfemoral; TCav, transcaval; TBra, transbrachiocephalic; Y, years; STS, Society of Thoracic Surgery score.

The TCav and TBra groups were excluded from this Kaplan–Meier analysis due to the very small number of patients (<30) treated via these access routes.

The cause of death was a cardiovascular event in 87% of cases, and a stroke in 47%.

The 30-day stroke rate was 4.3%: all were ischemic, and 40% of them were disabling. On multivariate analysis (Supplementary Table S2), prior stroke (HR 2.78, *p* = 0.028) and prior myocardial infarction (HR 2.69, *p* = 0.027) were independent predictors for stroke after TAVI, while there was a non-significant tendency for atrial fibrillation (HR 2.23, *p* = 0.06). After adjusting for confounding factors, P-Tax, but not S-TAx or PTA-TF, was associated with an increased risk of overall, but not disabling, stroke compared to TCar (adjusted OR: 4.21; 95%CI: 1.129–15.747; *p* = 0.003—adjusted OR: 4.51; 95%CI: 0.586–34.680; *p* = 0.147, respectively). There were no significant differences in the adjusted risk of overall or disabling stroke between P-TAx and S-TAx, or between PTA-TF and P-TAx or S-TAx (Table 4).

**Table 4:** Adjusted risks of adverse events stratified by access route.

Event	Unadjusted odds ratio	*p*-value	Adjusted odds ratio	*p*-value
	(95% confidence interval)		(95% confidence interval)	
Primary endpoint				
P-TAx vs. TCar	2.91 [1.282–6.590]	0.011	0.61 [0.278–1.337]	0.217
S-TAx vs. TCar	1.87 [0.674–5.239]	0.227	0.79 [0.313–2.014]	0.628
P-TAx vs. S-TAx	1.54 [0.624–3.831]	0.346	0.76 [0.282–2.087]	0.604
P-TAx vs. PTA-TF	1.27 [0.535–3.052]	0.581	0.67 [0.255–1.778]	0.425
S-TAx vs. PTA-TF	0. 82 [0.284–2.402]	0.726	0.87 [0.296–2.596]	0.813
PTA-TF vs. TCar	2.27 [0.842–6.138]	0.104	0.90 [0.375–2.186]	0.825
Stroke				
P-TAx vs. TCar	2.79 [0.844–9.267]	0.092	4.21 [1.129–15.747]	0.003
S-TAx vs. TCar	3.67 [1.008–13.416]	0.048	4.21 [0.940–18.908]	0.060
P-TAx vs. S-TAx	0.76 [0.260–2.218]	0.616	0.99 [0.285–3.501]	1.000
P-TAx vs. PTA-TF	1.12 [0.335–3.777]	0.848	1.07 [0.305–3.778]	0.910
S-TAx vs. PTA-TF	1.47 [0.400–5.463]	0.556	1.07 [0.253–4.563]	0.921
PTA-TF vs. TCar	2.48 [0.605–10.209]	0.206	3.92 [0.890–17.273]	0.007
Disabling stroke				
P-TAx vs. TCar	1.81 [0.298–10.974]	0.518	4.51 [0.586–34.680]	0.147
S-TAx vs. TCar	2.35 [0.325–16.991]	0.397	2.79 [0.212–36.751]	0.434
P-TAx vs. S-TAx	0.77 [0.125–4.705]	0.777	1.61 [0.157–16.533]	0.686
P-TAx vs. PTA-TF	0.74 [0.121–4.525]	0.744	0.86 [0.135–5.520]	0.878
S-TAx vs. PTA-TF	0.96 [0.131–7.000]	0.968	0.53 [0.046–6.251]	0.619
PTA-TF vs. TCar	2.44 [0.338–17.689]	0.375	5.21 [0.609–44.542]	0.131
Major vascular complication				
P-TAx vs. TCar	1.81 [0.298–10.974]	0.518	2.02 [0.314–13.076]	0.457
S-TAx vs. TCar	1.16 [0.103–12.984]	0.904	1.39 [0.117–16.515]	0.794
P-TAx vs. S-TAx	1.56 [0.159–15.246]	0.702	1.45 [0.148–14.309]	0.746
P-TAx vs. PTA-TF	0.28 [0.066–1.221]	0.091	0.26 [0.060–1.146]	0.075
S-TAx vs. PTA-TF	0.18 [0.020–1.596]	0.124	0.18 [0.020–1.592]	0.123
PTA-TF vs. TCar	6.37 [1.208–33.607]	0.029	7.71 [1.367–43.554]	0.021
Major bleeding				
P-TAx vs. TCar	4.50 [1.451–13.995]	0.009	7.91 [2.193–28.589]	0.001
S-TAx vs. TCar	1.76 [0.386–8.083]	0.463	1.08 [0.109–10.707]	0.945
P-TAx vs. S-TAx	2.55 [0.710–9.157]	0.151	7.31 [0.933–57.339]	0.058
P-TAx vs. PTA-TF	3.72 [0.824–16.839]	0.087	3.83 [0.827–17.759]	0.085
S-TAx vs. PTA-TF	1.46 [0.237–8.992]	0.682	0.52 [0.045–5.984]	0.603
PTA-TF vs. TCar	1.20 [0.216–6.746]	0.828	2.06 [0.342–12.453]	0.428
Overall mortality				
P-TAx vs. TCar	0.79 [0.219–2.859]	0.721	1.75 [0.408–7.571]	0.448
S-TAx vs. TCar	0.76 [0.151–3.881]	0.747	1.78 [0.299–10.670]	0.523
P-TAx vs. S-TAx	1.03 [0.185–5.769]	0.970	0.98 [0.170–5.673]	0.985
P-TAx vs. PTA-TF	2.01 [0.221–18.331]	0.534	2.28 [0.243–21.431]	0.469
S-TAx vs. PTA-TF	1.94 [0.172–21.938]	0.589	2.32 [0.200–26.912]	0.499
PTA-TF vs. TCar	0.39 [0.046–3.323]	0.391	0.76 [0.083–7.119]	0.817

P-TAx, percutaneous transaxillary; S-TAx, surgically-assisted transaxillary; TCar, transcarotid, PTA-TF, percutaneous transluminal angioplasty-assisted transfemoral; TCav, transcaval; TBra, transbrachiocephalic.

The rate of overall and disabling stroke was not statistically different among patients with than in those without CEPD (9.5 vs. 3.9%, *p* = 0.09 and 4.7 vs. 1.4%, *p* = 0.15).

Main access site-related major vascular complications occurred in 3% of cases in the total cohort and in 18% of cases in the TCav group (*p* = 0.013). PTA-TF was associated with a higher adjusted risk compared to TCar (adjusted OR: 7.71; 95%CI: 1.367–43.554), with no significant differences compared to the P-TAx or S-TAx groups.

In the P-TAx group, echo-guided puncture (86% of cases) had no significant impact on major vascular complications (1.5% vs. 4.5%, *p* = 0.37) or major bleeding (9% vs. 4.5%, *p* = 0.69).

Major bleeding occurred in 4% of cases, with a higher adjusted risk in the P-TAx group compared to the TCar group (adjusted OR: 7.91; 95%CI: 2.193–28.589).

Acute kidney injury ≥2 occurred in 7% of patients, with a significantly higher rate in the TCav group (29%, *p* < 0.0001).

BMI has no impact on the occurrence of adverse events.

There was a 10% rate of new pacemaker implantation, which was similar between the groups.

Device success was achieved in 90% of patients, with a significantly lower rate in the TBra group (74%, *p* < 0.0001). Indeed, a high rate of at least moderate aortic regurgitation (22%) led to poor valve performance, which was significantly lower with the TBra access route.

Since the inclusion period spansed 15 years, a stratified analysis by period (2009–2014, 2015–2019, 2020–2024) was performed to assess the impact of TAVI technology and patient's profile on the outcome. Surprisingly, we did not observe any significant difference in baseline characteristics between the three periods of time (Supplementary Table S3). Access routes were significantly different with more S-TAx approach in 2009–2014 and more P-TAx and PTA-TF in 2020–2024, while TCar remained stable overtime. The outcome of patient was similar between the 3 periods of time with no difference in the rate of adverse events overtime.

## 4 Discussion

The study's most important findings were:

1. Among patients with hostile iliofemoral anatomy, TAVI using various endovascular transarterial access route is efficient and safe, with 30-day overall and event-free survival rates of 97% and 91%, respectively, which were similar between groups.
2. The TCar access route exhibited the lowest stroke rate, while P-TAx was associated with a higher adjusted risk of overall, but not disabling, stroke compared to TCar.
3. PTA-TF provided the shortest hospital stay and lowest 30-day mortality (1.3%), and was not associated with an increased risk of death or stroke compared to TCar.

### 4.1 Efficiency and safety of TAVI in case of hostile iliofemoral anatomy

Among patients with severe peripheral arterial disease that precludes TF TAVI, series have reported the feasibility of alternative endovascular access routes, providing better results than the transthoracic approach, but with a relatively high rate of major adverse events compared with regular TF TAVI (2–5).

The Hostile registry (14) reported a 30-day mortality rate of 4.4% for both TF or other transarterial access among patients with STS scores of 5.9 and 5.3, respectively. In our series, the 30-day mortality rate was only 2.8%, with a relatively similar STS score of 5.3, with no differences between access routes. The causes of death were primarily cardiovascular and procedure-related, with most occurring during the index hospitalization (87% of cases).

Technical success was achieved in 95% of cases, device success in 90%, and the 30-day event-free survival rate was 91%, thereby confirming the high feasibility of TAVI via TCar, S-TAx, P-Tax, and PTA-TF despite unfavorable anatomy. Although TCav was technically feasible, it had a high rate of periprocedural vascular complications (18%; *p* = 0.01 vs. the total cohort) and lower technical success than PTA-TF (82% vs. 93%; *p* = 0.009).

TBra had a very high technical success rate (96%), but a significantly higher rate of at least moderate aortic regurgitation, resulting in a significantly lower device success rate compared to other access routes (74% vs. 90% in the total cohort; *p* < 0.0001).

In the general population of TF TAVI, the risk profile of patients moved from high at the early days to low risk in the current era. But, this shift of risk profile was not observed during the 15 years of the NORTHOSTAVI registry, including patients with hostile iliofemoral anatomy and severe peripheral arterial disease, undergoing TAVI with a similar outcome overtime. The fact that patients included during the period 2020–2024 comprised for 72% of the total cohort, may mitigate changes in profile and outcome overtime. Moreover, the risk profile of those TAVI candidates with peripheral arterial disease is so severe, that it could remain relatively stable between the year 2009 and 2024.

Access routes were more often S-TAx in the period 2009–2014 and P-TAx and PTA-TF in 2020–2024, while transcarotid remained stable overtime. The more recent development of IVL for peripheral disease and the improvement of the device's profile explain probably this shift from S-TAx to P-TAx and PTA-TF.

### 4.2 Stroke rate by access route

The stroke rate has been shown to be high among non-TF TAVI patients, mainly due to their higher-risk profile and hostile vascular anatomy (13, 14, 16). Despite advances in TAVI techniques, stroke remains the most feared complication, which can be caused by dislodgment of calcified debris, aortic valve tissue, or a transient reduction in cerebral blood flow during the procedure.

Although a higher risk of cerebrovascular events was anticipated with the TCar access route, Beurtheret et al. (2) reported similar stroke rates with TCar and TAx (3.6% vs. 2.9%, *p* = NS), and Chamandi et al. (9) demonstrated similar stroke rates with TCar and transthoracic TAVI (2.1% vs. 3.5%, *p* = NS). In our series, the 30-day overall stroke rate was 4.3% in the total cohort and 2% in the TCar group. Patient selection, operator experience, and procedural optimization, including monitoring of cerebral perfusion pressure, can explain this relatively low incidence of cerebrovascular events, but still have disastrous consequences.

Indeed, strokes accounted for half of the deaths in our NORTHOSTAVI registry. TCar had the lowest stroke rate, and P-TAx, but not PTA-TF, was associated with a higher adjusted risk of stroke compared to TCar. In the Hostile registry (14), Palmerini et al. reported higher 1-year stroke/transient ischemic attack rates with the alternative transarterial route than with the TF route, with the transaxillary approach comprising for 92% of the alternative transarterial group. On the other hand, Van Wely et al. (10) stated that as a default approach in patients with an anatomy compatible with TF access, transaxillary TAVI provided a similar outcome as TF, with reported stroke rates of 2% and 3%, respectively (*p* = 0.19). In the present registry, procedural aspects could have had an impact on the stroke rate, especially the use of pre- and post-ballooning, which were reported as risk factors for stroke after TAVI (17). Pre-dilatation was used significantly less often in the TCar group (27% vs. 60% in the total cohort and 82% in P-TAx), which is probably related to the different types of valves implanted according to access route: In the TCar group, 89% of implants were Corevalve/Evolut and 9% were Sapien, which do not require pre-ballooning in most cases. On the other hand, pre-dilatation is common with the Portico/Navitor platform, which was used in 49% of P-TAx cases, but only 2% of TCar cases.

Cerebral embolic protection devices (CEPDs) have been developed to decrease embolic brain injury and stroke during TAVI. Results of metanalysis of randomized trials shown a reduction of ischemic brain lesions with less evidence on the clinical events (18). Recently, a large US registry (19) and PROTECTED TAVR study (20) demonstrated that the use of CEPD was associated with a lower risk of major but not of overall stroke. Those studies involved only TF access route. Alternatives endovascular transarterial access routes were not included in randomized trials, while it is well known that patients with severe peripheral arterial disease undergoing a non-TF TAVI are at higher risk of stroke, and would be an ideal target group to assess such preventive strategies.

From a technical point of view, placement of the Sentinel device (Boston Scientific Corp., Boston, US) by the right radial artery is not always possible due to the poor vascular anatomy of the arm or the aortic arch. For patients undergoing TCar TAVI, deployment of the filter in the left carotid is not possible. The device cannot be deployed during a right TAx or a TBra TAVI. Devices like TriGUARD (Keystone Heart, Tampa,US), deployed by the femoral artery would be more appropriate for those alternative access. In the NORTHOSTAVI registry, the Sentinel CEPD was used in 7.9% of patients with no significant impact on overall and disabling stroke.

A new strategy woud be applied to protect patients undergoing a left P-TAx TAVI, a very high stroke risk group, using a Sentinel CEPD deployed by the right radial artery and a large bore sheath inserted in the left axillary artery covering the left vertebral artery, left in place at the ostium of the left subclavian artery during the exchanges of material. The impact of this new technique on stroke rate after P-TAx TAVI would be evaluated in future trials.

### 4.3 Transfemoral access route facilitated by peripheral angioplasty

As the TF access is the technique of choice, supported by randomized trials showing the superiority of TF TAVI over SAVR (1, 21, 22), techniques for preparing iliofemoral vessels are appealing in order to avoid a non-TF procedure despite hostile anatomy. IVL has been shown to be feasible in small series (12, 23): by fracturing the calcium within the vessel wall using acoustic waves, it improves vessel compliance and ultimately allows for the introduction of a large-bore delivery sheath. This relatively short balloon-assisted technique seems more appropriate for concentric and focal calcifications.

In cases of more diffuse disease and/or the presence of asymmetric calcifications with nodules, orbital atherectomy would be a more appropriate technique, as it allows for debulking of the vessel (24). In the present registry, 14% of patients with hostile anatomy underwent PTA-TF, which was primarily IVL-assisted, with a 93% technical success rate. A higher rate of vascular complications is expected in this population compared to favorable iliofemoral access. Major vascular complications were reported in approximately 15%–20% of patients with hostile vascular access (14), as opposed to less than 5% of patients with regular anatomy (21, 22). In our series, the rate of major vascular complications was 3%. PTA-TF was associated with a higher adjusted risk of vascular complications than TCar, but not than P-TAx or S-TAx. These vascular complications were mainly alleviated through endovascular stenting, and PTA-TF was not associated with an increased risk of stroke or death compared to TCar. Patients undergoing PTA-TF had the shortest hospital stay and the lowest 30-day mortality (1.3%), while avoiding general anesthesia, surgical cut-down, and surgical closure.

## 5 Conclusion

The NORTHOSTAVI registry, which included patients with hostile iliofemoral anatomy, showed that TAVI using various endovascular transarterial access route was efficient and safe, with a technical success rate of 95% and 30-day overall and event-free survival rates of 97% and 91%, respectively, similar between groups. At 30 days, TCar exhibited the lowest stroke rate (2%), P-TAx was associated with an increased adjusted risk of overall stroke while PTA-TF was associated with the shortest hospital stay and the lowest 30-day mortality rate (1.3%), with no increased adjusted risk of death or stroke, compared to TCar.

In patients with hostile iliofemoral anatomy, the alternative TAVI access route should be selected based on patient characteristics and local expertise.

## 6 Impact on daily practice

In patients with hostile iliofemoral anatomy, TAVI via an alternative access route is efficient and safe (30-day overall and event-free survival rates of 97% and 91%, respectively, similar between groups), with a higher adjusted risk of stroke and major bleeding for P-TAx and of major vascular complications for PTA-TF compared to TCar.

## 7 Limitations

This report is a retrospective analysis of prospectively acquired data, which is subject to limitations inherent in the study design. The selection of patients was not random. As a consequence, a bias of patient selection by access route remains possible and may not have been fully eliminated by adjusting for confounding factors in the statistical comparison between groups.

The use of pairwise comparisons between access route using separate multivariable models may overstate statistical significance of the results.

The choice of the access route was selected by the operators, based on their preference and expertise and on the availability of local resources. Not all alternative techniques were used in all centers: TBra was only used in one center, while five centers only used one alternative access.

Due to the small number of patients, TCav and TBra could not be included in the Kaplan–Meier analysis or the multivariate logistic regression analysis. Nevertheless, we reported observational data on these interesting groups of patients.

This was a multicenter registry without a core lab or clinical event committee; each center reported its data according to the VARC-3 criteria.

The detailed reason for unsuitability of transfemoral access route was not specified by the centers.

Since this registry collected data from 14 centers in Belgium, Denmark, and the Netherlands, our data may not be generalizable to all patients in all centers.

Since patients were included in a 15-year period of time, with variation of patient's profile, improvement of operator's experience and devices, the results of the present study may not be generalized to the current TAVI population.

## Data Availability

The raw data supporting the conclusions of this article will be made available by the authors, without undue reservation.
