# Supplementary material for: The NORTh Europe HOStile access TAVI (NORTHOSTAVI) registry
Source: Front Cardiovasc Med. 2025 Sep 26;12:1674218. doi: 10.3389/fcvm.2025.1674218 (PMC12513205; doi:10.3389/fcvm.2025.1674218)
Supplement: Supplementary file 4 [file Supplementaryfile1.pdf]

Ethical committees of each center

Ethical committee of:

Cliniques universitaires Saint-Luc Brussels, Belgium
The Heart Center, Righospitalet, Copenhagen, Denmark
CHU Saint-Pierre, Brussels, Belgium
Gasthuisberg, UZ Leuven, Leuven, Belgium
UZ Antwerpen, Antwerpen, Belgium
AZ Sint-Jan Campus, Brugge, Belgium
CHR Citadelle, Liège, Belgium
Centre Hospitalier Universitaire de Charleroi, Charleroi, Belgium
Hartcentrum Aalst, AZORG, Aalst, Belgium
Hartcentrum AZ Delta, Roeselare, Belgium
CHU UCL Namur- Mont Godinne, Yvoir, Belgium
Jessa Ziekenhuis, Hasselt, Belgium
UZ Brussel Jette, Brussels, Belgium
University Medical Center, Groningen, the Netherlands
